# Supplementary material for: First Reported Circulation of Equine Influenza H3N8 Florida Clade 1 Virus in Horses in Italy
Source: Animals (Basel). 2024 Feb 12;14(4):598. doi: 10.3390/ani14040598 (PMC10886299; doi:10.3390/ani14040598)
Supplement: Supplementary file 1 [file animals-14-00598-s001.zip › animals-2813906-supplementary.pdf]

Table S1 Accession numbers of the gene sequences included in the phylogenetic analysis for HA and NA trees were obtained from the NCBI database (unavailable sequences are described as n.a., not available).

Origin	Year	HA fragmen Accesion Number	NA fragmen Accesion Number
California	1980	CY028812.1	CY028814.1
Miami	1963	CY028836.1	CY028838.1
Rome	1991	n.a.	CY032367.1
Berlin	1989	CY032413.1	CY032415.1
Spain	2007	CY075851.1	CY075852.2
Ohio	2003	DQ124192.1	DQ124168.1
Bari	2005	EF117330.1	n.a.
Richmond	2007	FJ195395.3	KF559336.1
Lincolnshire	2007	FJ195398.2	n.a
Lincolnshire	2007	n.a.	KF049175.1
Lichtenfeld	2012	n.a.	KF049191.1
Perthshire	2009	n.a.	KF049193.1
Devon	2011	n.a.	KF049194.1
Shropshire	2010	n.a.	KF049195.1
Dorset	2009	n.a.	KF049196.1
Lanarkshire	2009	n.a.	KF049197.1
NewMarket	2003	FJ375213.1	n.a.
Almaty	2007	GU953266.2	n.a.
Donegal	2007	JN222934.1	n.a.
Meath	2007	JN222935.2	MG586816.1
Kildare	2007	JN222936.1	n.a.
Kildare	2010	n.a.	MG586819.1
Kildare	2012	n.a.	MG586821.1
Kildare	2014	n.a.	MG586826.1
Down	2008	JN222937.1	MG586817.1
Donegal	2009	JN222938.1	n.a.
Carlow	2009	JN222939.1	n.a.
Carlow	2011	n.a.	MG586820.1
Limerick	2010	JN222940.1	n.a.
Limerick	2010	n.a.	MG586818.1
Grosbois	2009	JX091759.2	KY241349.1
Kyonggi	2011	JX844146.2	JX844148.2
East Renfrewshire	2011	n.a.	KF049172.1
Worcestershire	2012	n.a.	KF049174.1
Xuzhou	2013	KF806985.1	KF806987.1
Pulawy	2008	KT429521.1	n.a.
Pulawy	2018	n.a	MZ364006.1
France	1986	KY241305.1	KY241337.1
Grosbois	2003	KY241313.1	KY241345.1
Belfond	2009	KY241314.1	KY241350.1
Cagnes-sur-Mer	2011	KY241315.1	KY241346.1

Neuville-Pres-Sees	2011	KY241316.1	KY241347.1
Cambremer	2012	KY241317.1	KY241351.1
Ain	2014	KY241318.1	n.a.
Gironde	2014	KY241319.1	KY241352.1
Saone-et-Loire	2015	KY241320.1	KY241353.1
Kentucky	1994	L39914.1	n.a.
Wexford	2014	MG586802.1	n.a.
Meath	2014	MG586804.1	MG586823.1
Tipperary	2014	MG586805.1	n.a.
Kilkenny	2012	n.a.	MG586822.1
Kilkenny	2014	MG586811.1	MG586828.1
Santiago	2018	MH346720.1	MH346583.1
Paris	2018	MK501760.1	n.a.
Pas-de-Calais	2018	MK501761.1	MK501801.1
Ardennes	2018	MK501762.1	n.a.
Calvados	2019	MK501763.1	MK501802.1
South Africa	2003	ON797670.1	ON797672.1
United Kingdom	2019	OQ379795.1	OQ379797.1
Brescia	2019	OP926929.1	OP926930.1

Table S2: Marginal likelihood values of the arithmetic and harmonic means obtained in the four runs and the overall mean values.

HA Run	Arithmetic Mean	Harmonic Mean
1	-2687.89	-2718.41
2	-2689.06	-2721.10
3	-2689.48	-2716.55
4	-2687.17	-2717.93
Total	-2688.00	-2719.83

Table S3: Marginal likelihood values of the arithmetic and harmonic means obtained in the four runs and the overall mean values.

NA Run	Arithmetic Mean	Harmonic Mean
1	-2846.15	-2881.48
2	-2847.72	-2888.82
3	-2846.36	-2874.76
4	-2845.45	-2874.03
Total	-2846.14	-2887.44
